# Construction and validation of a nomogram for predicting survival in elderly patients with severe acute pancreatitis: a retrospective study from a tertiary center

**DOI:** 10.1186/s12876-024-03308-6

**Published:** 2024-07-08

**Authors:** Qingcheng Zhu, Mingfeng Lu, Bingyu Ling, Dingyu Tan, Huihui Wang

**Affiliations:** Department of Emergency Medicine, Northern Jiangsu People’s Hospital Affiliated to Yangzhou University, Yangzhou, 225001 China

**Keywords:** Severe acute pancreatitis, Elderly patients, Mortality, Prediction model, Nomogram

## Abstract

**Purpose:**

There is a lack of adequate models specifically designed for elderly patients with severe acute pancreatitis (SAP) to predict the risk of death. This study aimed to develop a nomogram for predicting the overall survival of SAP in elderly patients.

**Methods:**

Elderly patients diagnosed with SAP between January 1, 2017 and December 31, 2022 were included in the study. Risk factors were identified through least absolute shrinkage and selection operator regression analysis. Subsequently, a novel nomogram model was developed using multivariable logistic regression analysis. The predictive performance of the nomogram was evaluated using metrics such as the receiver operating characteristic curve, calibration curve, and decision curve analysis (DCA).

**Results:**

A total of 326 patients were included in the analysis, with 260 in the survival group and 66 in the deceased group. Multivariate logistic regression indicated that age, respiratory rate, arterial pH, total bilirubin, and calcium were independent prognostic factors for the survival of SAP patients. The nomogram demonstrated a performance comparable to sequential organ failure assessment (*P* = 0.065). Additionally, the calibration curve showed satisfactory predictive accuracy, and the DCA highlighted the clinical application value of the nomogram.

**Conclusion:**

We have identified key demographic and laboratory parameters that are associated with the survival of elderly patients with SAP. These parameters have been utilized to create a precise and user-friendly nomogram, which could be an effective and valuable clinical tool for clinicians.

## Introduction

Acute pancreatitis (AP), characterized by rapid progression, multiple organ failure, and high mortality, is a common inflammatory disease of pancreas worldwide [1]. While most cases are mild, with a global incidence ranging from 8 to 50 cases per 100,000 individuals annually [2], a significant proportion of patients (15–35%) will experience a severe episode with mortality rates reaching up to 30% [3]. Studies have shown that patients diagnosed with severe acute pancreatitis (SAP) may experience improved outcomes through enhanced monitoring, prompt aggressive fluid resuscitation, and early enteral feeding [4, 5]. Early diagnosis and accurate assessment of disease severity are essential for timely intervention.

In recent years, various scoring systems have been used for risk stratification in AP, including sequential organ failure assessment (SOFA), Acute Physiology and Chronic Health Evaluation II (APACHE II), and Ranson [6]. However, these systems have drawbacks such as complexity, lack of convenience, and suboptimal accuracy. Recent studies have suggested that C-reactive protein [7], red blood cell distribution width [8], and D-dimer [9] may serve as potential predictors of hospital mortality in SAP. Nevertheless, there is inconsistency in the thresholds of these individual predictors across different studies.

A nomogram is a useful mathematical tool used to predict certain outcomes, such as disease progression or mortality, based on various key parameters [10]. While some studies have created nomograms to predict in-hospital mortality of AP using critical care databases [11, 12], their main limitations were small sample sizes and lack of external validation. These studies have identified age as an independent risk factor for death in AP patients. According to Gardner et al., individuals aged 70 years and older are identified as an independent risk factor for mortality in patients with SAP [13]. While AP presents similarly in both younger and older patients, certain characteristics are unique to the elderly population. In cases of acute necrotizing pancreatitis, elderly patients face a higher likelihood of complications, including multisystem failure [14]. As a result, it is recommended that these patients receive closer monitoring and more aggressive treatment. However, there is a lack of systematic models specifically designed for elderly patients with SAP to predict in-hospital mortality.

The aim of this study was to identify risk factors capable of predicting hospital mortality in elderly patients with SAP and to construct a practical and efficient nomogram model. This model aims to equip clinicians with valuable personalized intervention information in advance.

## Methods

### Study design and eligibility

This retrospective study was carried out in an 81-bed intensive care unit (ICU) at a tertiary teaching hospital in Jiangsu Province, China. The research adhered to the ethical principles outlined in the amended Declaration of Helsinki. Approval for the study was obtained from the Institutional Ethics Committee of Northern Jiangsu People’s Hospital (No. 2,023,035), and written informed consent was not required due to the retrospective nature of the study. Patient information was de-identified and anonymized prior to analysis.

**Fig. 1:**
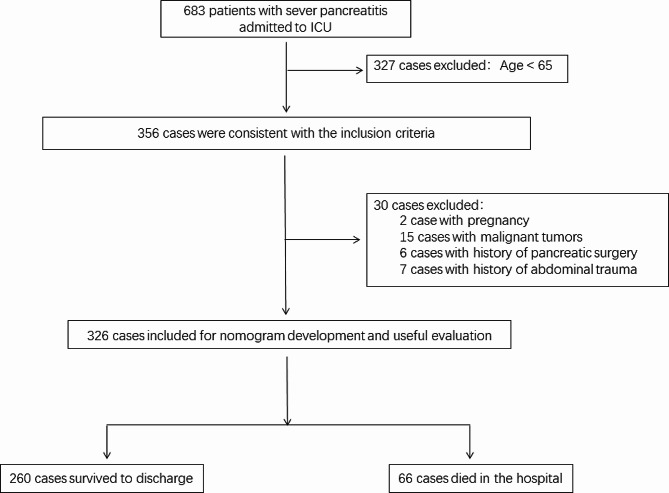
Flow chart of patient enrollment. ICU, intensive care unit

The study population consisted of patients admitted to the ICU between January 1, 2017, and December 31, 2022, with severe pancreatitis. Patients who were transferred to the ICU due to worsening of their condition during hospitalization were excluded from the analysis. The diagnosis of AP was confirmed if at least two of three criteria were met: abdominal pain, elevated levels of amylase or lipase exceeding three times the upper limit of normal, or abdominal imaging findings consistent with AP, according to the 2012 revised Atlanta criteria [15]. SAP was defined as the presence of persistent organ failure (> 48 h) in patients. Organ failure was defined by a Marshall score of ≥ 2, indicating that at least one organ system (respiratory, cardiovascular, renal) must be affected. The inclusion criteria for this study required participants to be aged 65 years or older and have complete laboratory parameters and clinical data available within 24 h of admission. Exclusion criteria included pregnancy, malignant tumors, history of pancreatic surgery, and history of abdominal trauma. The patients were then divided into two groups: those who survived (*n* = 260) and those who died (*n* = 66). The flow chart illustrating patient selection can be found in Fig. 1.

### Data collection

The data were collected from the electronic medical record archives and included demographic features (gender, age), height, weight, past medical history, as well as vital signs on admission including respiratory rate (RR), heart rate (HR), systolic blood pressure (SBP), and diastolic blood pressure (DBP). Additionally, the SOFA scores on admission were also recorded. The laboratory indices extracted for analysis included red blood cells, white blood cells, platelets, neutrophils, lymphocytes, hemoglobin, hematocrit, total bilirubin, alanine aminotransferase, aspartate aminotransferase, serum sodium, serum potassium, serum calcium, glucose, creatinine, albumin, prothrombin time, activated partial thromboplastin time, triglycerides, cholesterol, serum amylase, serum lipase, and arterial blood gas analysis. The arterial blood gas analysis encompassed arterial pH, arterial oxygen partial pressure (PaO_2_), arterial carbon dioxide partial pressure (PaCO_2_), and lactic acid.

### Statistical analysis

The Kolmogorov-Smirnov test was used to test the normal distribution for measurement data. Normally distributed data were expressed as means ± standard deviation, and the skewed distributed data was reported as medians (quartiles). The two groups were compared using t tests or Mann-Whitney U tests. Categorical data were expressed as a percentage, using χ 2 or Fisher’s exact probability tests. The study employed the least absolute shrinkage and selection operator (LASSO) regression method to determine the key predictors for hospital mortality. Following this, a nomogram was developed using a multivariate logistic regression model with the identified variables.

The area under the receiver operating characteristic curve (AUC) and Harrell’s concordance index (C-index) were utilized to assess the predictive accuracy of the developed nomogram. A calibration curve was used to evaluate the concordance between the predicted probabilities and the observed outcomes. The net reclassification improvement (NRI) was utilized for comparing the predictive accuracy of the nomogram and SOFA, while the integrated discrimination improvement (IDI) was employed to determine the efficacy of the enhancements. The clinical relevance of the prediction model was evaluated through decision-curve analysis (DCA). All statistical analyses were conducted using R software (version 3.6.1, CRAN) and SPSS (version 24.0, Chicago, IL). A *P*-value < 0.05 means significant statistical difference.

## Results

### Baseline characteristics and outcomes

Out of the 356 SAP patients who met the inclusion criteria during the study period, 30 were excluded for various reasons (2 patients were pregnant, 15 had advanced tumors, 6 had a history of pancreatic surgery, and 7 had a history of abdominal trauma). Ultimately, 326 patients were selected, with 260 in the survival group and 66 in the deceased group (Fig. 1). The in-hospital mortality rate in our study was 20.25%.

Among all the characteristics, age, cholelithiasis, RR, SBP, DBP, SOFA, platelet, total bilirubin, serum calcium, creatinine, albumin, prothrombin time, activated partial thromboplastin time, and arterial pH differed significantly between the two groups (*P* < 0.05). Other baseline clinicopathological data were similar between the two groups, as indicated in Table 1.

**Table 1:** Baseline characteristics of selected patients

Characteristics	Survived(*n* = 260)	Died(*n* = 66)	*p* value
Male, n (%)	140(53.8)	33(50.0)	0.576
Age, years	75(69–82)	79(72–86)	0.012
Height (cm)	168.8(165.1-172.6)	167.6(162.6-175.1)	0.598
Weight (kg)	79.9(68.4–88.6)	83.5(67.0-97.1)	0.218
Comorbidities, n (%)			
COPD	7(2.69)	0(0)	0.178
Diabetes mellitus	77(29.6)	20(30.3)	0.913
Coronary artery disease	55(21.2)	15(22.7)	0.781
Hypertension	130(50.0)	35(53.0)	0.660
Cholelithiasis	131(50.4)	24(36.4)	0.042
Hyperlipemia	49(18.8)	9 (13.6)	0.323
Vital signs			
Respiratory rate (/min)	19(19–20)	20(19–22)	0.001
Heart rate (/min)	40(40–86)	58(40–107)	0.129
Systolic blood pressure (mmHg)	122(122–124)	122(114–122)	0.024
Diastolic blood pressure (mmHg)	65(54–71)	65(59–88)	0.011
SOFA	4(3–6)	9(6–11)	< 0.001
Laboratory data			
Red blood cell (*10^12^)	4.03(3.57–4.50)	3.84(3.26–4.48)	0.118
White blood cell (*10^9^)	12.75(9.40-17.85)	15.30(9.60–18.40)	0.349
Platelet (*10^9^)	225 (179–303)	218(125–284)	0.039
Neutrophil (*10^9^)	4.1(3.0-5.8)	4.3(3.2–5.7)	0.841
Lymphocyte (*10^9^)	9.0(5.0-14.2)	7.6(4.0–11.0)	0.084
Hemoglobin (g/L)	121(106–137)	117(101–136)	0.204
Hematocrit (%)	36.6(31.9–40.6)	35.6(30.7–41.8)	0.580
Total bilirubin (mg/dL)	1.1(0.6–2.6)	1.3(0.8–4.6)	0.050
Alanine aminotransferase (U/L)	54.5(22.5-171.5)	61(24.0-170.0)	0.695
Aspartate aminotransferase (U/L)	67.0(28.5–170.0)	74.0(31.0-225.0)	0.379
Sodium (mmol/L)	139.0(136.5–142.0)	139.0(136.6-151.2)	0.899
Potassium (mmol/L)	3.9(3.6–4.4)	4.1(3.8–4.8)	0.124
Calcium (mmol/L)	2.13(2.00-2.28)	1.99(1.85–2.20)	0.002
Glucose (mg/dL)	132(106–182)	146(105–214)	0.281
Creatinine (mg/dL)	1.2(0.9–1.7)	1.3(1.0-2.2)	0.007
Albumin (g/dL)	3.2(2.7–3.8)	3.1(2.6–3.5)	0.024
Prothrombin time (seconds)	13.75(12.90-15.55)	15.05(13.30–17.50)	0.004
Activated partial thromboplastin time (seconds)	28.5(25.0-32.5)	30.7(27.3–64.4)	0.004
Triglyceride (mg/dL)	236 (115–255)	220(102–255)	0.512
Cholesterol (mg/dL)	170(135–200)	175(142–195)	0.640
Amylase (U/L)	225(69–591)	230(70–536)	0.500
Lipase (U/L)	256(49-1299)	249(55-1015)	0.493
Arterial pH	7.40(7.35–7.45)	7.33(7.21–7.40)	< 0.001
PaO_2_ (mmHg)	120(95–140)	114(84–153)	0.153
PaCO_2_ (mmHg)	40(35–42)	37(30–43)	0.063
Lactic acid (mmol/L)	2.9(1.5–3.2)	2.9()0.1.8–3.3	0.076

### Construction of a predictive nomogram

**Fig. 2:**
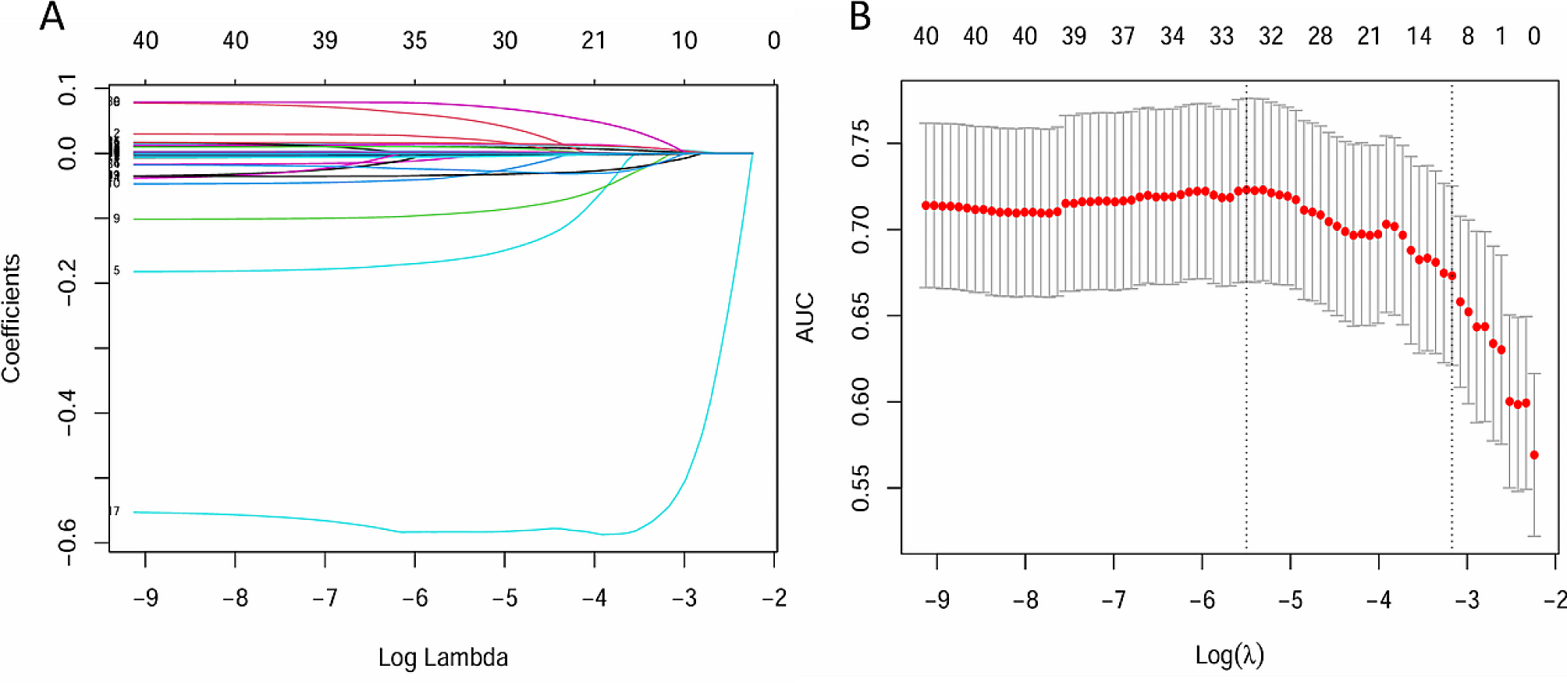
Identification of the risk factors of hospital mortality by LASSO regression (**A**) Least absolute shrinkage and selection operator coefficient profiles of the 41 variables. (**B**) Tuning parameter selection in the LASSO model used 10-foldcross validation via minimum criteria. The area under the receiver operating characteristic (AUC) curve was plotted versus log(λ). Dotted vertical lines were drawn at the optimal values by using the minimum criteria and the 1 standard error of the minimum criteria

**Fig. 3:**
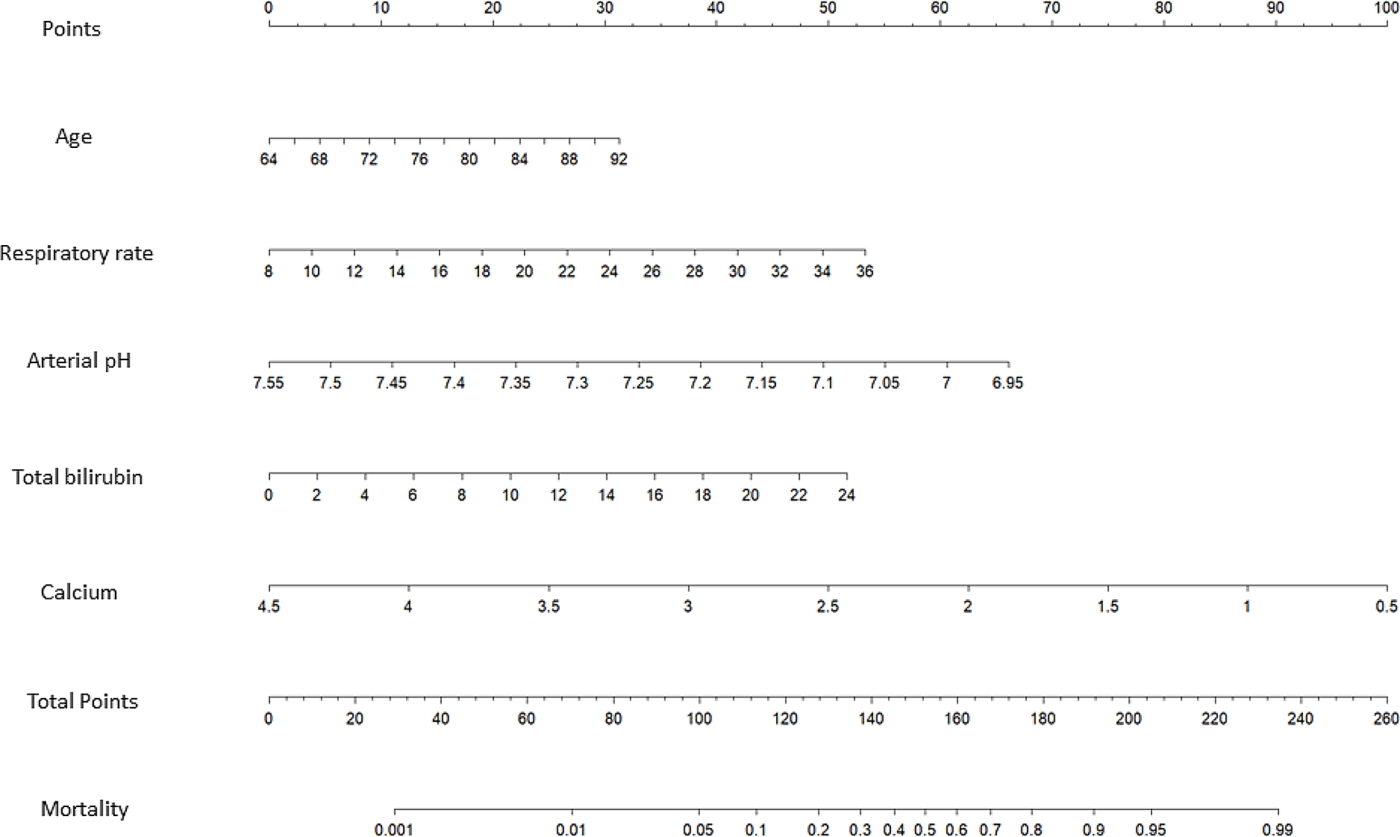
Predictive nomogram for the probability of hospital mortality in elderly SAP patients. SAP, severe acute pancreatitis

The variables identified were further analyzed using LASSO binary logistic regression with a lambda selected based on the 1 standard error criteria (Fig. 2A and B). A total of 12 independent risk factors associated with hospital mortality were identified, which included age, cholelithiasis, RR, SBP, arterial pH, lactic acid, platelet, neutrophil, total bilirubin, potassium, calcium, and albumin upon admission for patients who survived compared to those who did not. Subsequently, a multivariate logistic regression analysis was conducted on these 12 variables, leading to the development of a multi-factor risk model through the stepwise backward method (Table 2). Age, RR, arterial pH, total bilirubin, and calcium were then integrated to create a novel predictive nomogram (Fig. 3).

**Table 2:** Multivariate logistic regression analysis of the predictors for hospital mortality

Variables	OR	95%CI	*p* value
**Age**	3.083	1.707–5.568	< 0.001
**Cholelithiasis**	0.417	0.201–1.167	0.719
**Respiratory rate**	1.114	1.028–1.207	0.008
**Systolic blood pressure**	0.040	0.002–1.120	0.059
**Arterial pH**	0.680	0.523–0.883	0.004
**Lactic acid**	1.123	0.948–1.331	0.178
**Platelet**	0.762	0.502–1.157	0.202
**Neutrophil**	0.829	0.636–1.080	0.165
**Total bilirubin**	1.360	1.073–1.723	0.011
**Potassium**	1.504	0.833-2.000	0.125
**Calcium**	0.596	0.394–0.901	0.014
**Albumin**	0.576	0.321–1.034	0.065

### Evaluation and validation of the nomogram

**Fig. 4:**
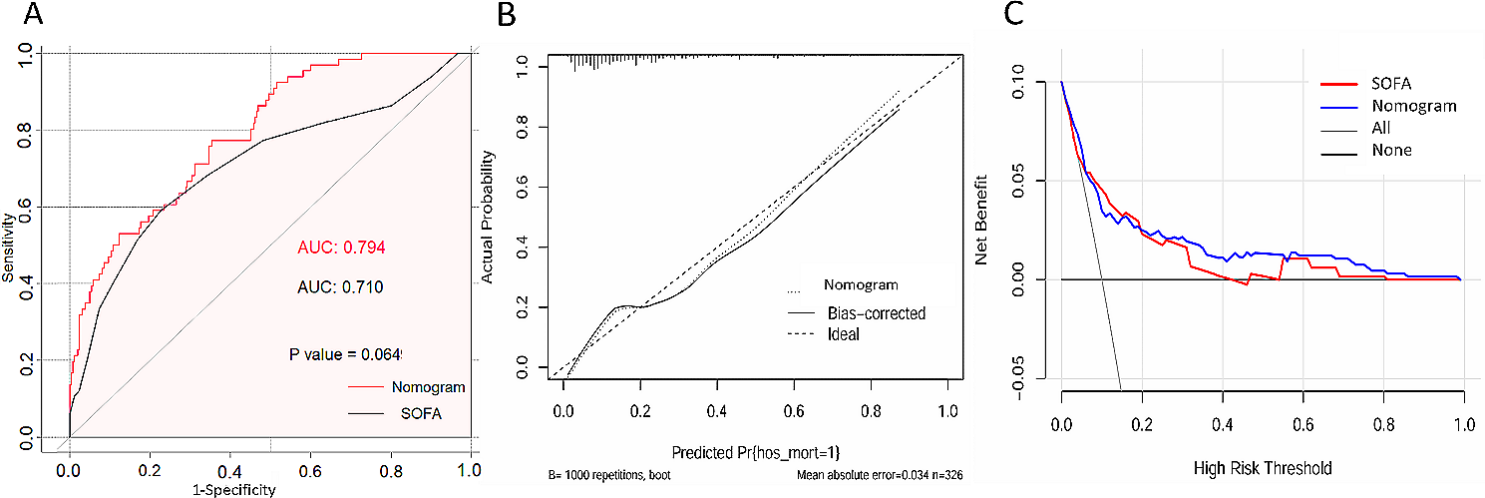
(**A**) The receiver operating characteristic curves. (**B**) The calibration curves. (**C**) The decision curves

The receiver operating characteristic (ROC) curve analysis was used to assess the diagnostic accuracy of the nomogram model. Upon visual examination of the AUC, the nomogram model demonstrated superior performance compared to SOFA score. However, the AUC values of nomogram and SOFA score were 0.794 and 0.710, respectively, with no statistically difference (*P* = 0.065, Fig. 4A). The parameters of the ROC curves at the nomogram cut-off point are shown in Table 3.

**Table 3:** Comparison of the nomogram model and SOFA for predicting the hospital mortality

Variables	C-index (95%CI)	Sensitivity	Specificity	IDI	NRI
**nomogram**	0.794(0.737–0.851)	0.646	0.773	0.030	-0.012
**SOFA**	0.710(0.633–0.787)	0.773	0.591	-	-

To validate the model’s performance internally, bootstrapping technique was utilized. the calibration plot visually demonstrates a strong agreement between the predicted and actual in-hospital mortality (Fig. 4B). In this study, the IDI was 0.030 (95% CI − 0.042 to 0.102), indicating no significant difference (*P* = 0.415) in performance between the nomogram model and SOFA score. Additionally, the NRI value was − 0.012 (95% CI − 0.177 to 0.154), with no significant difference (*P* = 0.891) (Table 3). Figure 4C illustrates the net benefit of using the nomogram model and SOFA score, showing several overlaps where the net benefit of the nomogram model was comparable to that of SOFA score. The DCA demonstrated that the nomogram exhibited a superior overall net benefit across a broad and practical range of threshold probabilities, as illustrated in Fig. 4C. Furthermore, clinical interventions guided by our nomogram showed a higher net benefit compared to the SOFA score when the threshold probability ranged from 0.2 to 0.8.

## Discussion

Most patients with AP will recover spontaneously without any organ failure, while approximately 20% of cases will progress to a severe form that is linked to multiple organ dysfunction syndrome, sepsis, and high mortality [16]. Age is a well-established factor associated with negative outcomes in AP patients, especially in the elderly [17]. To the best knowledge, this is the first study to develop a systematic nomogram specifically tailored for elderly patients with SAP. In our study, we identified 5 predictors, including age, RR, arterial pH, total bilirubin, and calcium, as independent prognostic factors for in-hospital mortality of SAP in elderly patients. The nomogram demonstrated excellent performance in predicting in-hospital mortality of SAP. Furthermore, our nomogram exhibited similar discrimination and clinical applicability when compared to the traditional SOFA system.

The current clinical evaluation indicators for the severity and prognosis of SAP include the SOFA score, APACHE II score, and Ranson score. SOFA and APACHE II are widely used prognostic tools in the ICU setting to predict severity and mortality [18]. However, a limitation of both SOFA and APACHE II is that they rely on numerous variables that are not routinely collected upon general hospital admission [19, 20]. On the other hand, the Ranson score necessitates 48 h of inpatient observation [21], leading to delayed triage and management. Some studies have indicated that CT images can diagnose and assess the severity of SAP, but these features typically manifest in abdominal CT scans of AP patients 48 h after onset [22], with limited predictive value within the first 24 h. The nomogram model stands out due to its simplicity and accuracy, requiring only five parameters that do not involve additional calculations and are easily accessible to clinicians. Apart from age, other risk factors can be modified through timely and aggressive treatment, which is crucial for enhancing patient outcomes.

Older age has long been recognized as a significant indicator of poor prognosis in AP [23], as evidenced by its inclusion in both the APACHE II score and Ranson score as a predictive factor. This association is likely attributed to the higher likelihood of comorbid conditions as age increases [24]. In elderly patients with limited organ reserve, there is a chronic inflammatory state present, leading to a higher incidence of adverse events [25]. Our research findings indicated that the hospital mortality rate for older patients with SAP was 20.25%, consistent with existing literature [26]. Additionally, age was identified as an independent risk factor in our study, underscoring the importance for clinicians to closely monitor older patients with poorer prognoses associated with advanced age.

There is substantial evidence supporting the significance of RR as a key physiological indicator in severely ill patients and in the worsening of clinical symptoms [27]. Acute respiratory failure (ARF) has been identified as the primary form of organ failure in both early and late stages of AP [1]. In a retrospective study of 813,120 hospitalized AP patients, Gajendran et al. found ARF in 21,415 cases (2.63%), with a corresponding mortality rate of 17% [28]. SAP can often be complicated by ARF, leading to increased mortality [29]. The study highlights the importance of RR as a critical prognostic factor for hospital mortality, emphasizing the need for clinicians to promptly identify the underlying causes of increased respiratory rate and provide personalized treatment.

The association between acidosis and an increase in multiple organ failure and mortality for intensive care patients has been well-documented. A study found that severe metabolic or mixed acidosis, defined by a plasma pH lower than 7.20, occurs in 6% of critically ill patients within the first 24 h in the ICU, and was linked to high mortality rates [30]. However, there is limited research of acidosis on predicting SAP. In a prospective study focusing on young and middle-aged patients, Sharma discovered that those with metabolic acidosis (pH < 7.35) had higher rates of organ failure, interventions, and mortality [31]. Our study further supports this by highlighting that arterial pH at presentation could be a valuable early indicator for predicting hospital mortality in elderly patients with SAP.

Total bilirubin was identified as a significant predictor for mortality in AP, a finding that had been supported by numerous studies [27]. Prior research has shown that in cases of AP, obstruction of the bile duct could impede the excretion of bile, leading to the accumulation of bilirubin in liver cells [32]. This accumulation disrupts the normal metabolic processes of the liver cells, ultimately resulting in their degeneration, necrosis, and impaired function. Elevated bilirubin levels are associated with a poor outcome [33]. Our study similarly revealed that total bilirubin serves as a risk factor for hospital mortality in elderly SAP patients.

Hypocalcemia is a crucial element in Ranson’s scoring system used to evaluate the severity of pancreatitis [34]. Research indicates that the level of calcium plays a significant role in both the exocrine functions of the pancreas and the pathological progression of AP. A study by Chhabra et al. found that patients with hypocalcemia during AP had a notably higher incidence of persistent organ failure, need for intervention, and mortality compared to those with normal serum calcium levels [35]. The suggested mechanisms for hypocalcemia in the early phase include autodigestion of mesenteric fat by pancreatic enzymes leading to the release of free fatty acids that form calcium salts, transient hypoparathyroidism, and hypomagnesemia [36]. Nonetheless, our study also identified calcium as an independent predictor of hospital mortality for elderly SAP patients.

This retrospective study investigated mortality risk factors in elderly patients with SAP and developed a nomogram for precise prediction of in-hospital mortality. The SOFA score has been identified as a valuable tool for predicting short-term mortality in critically ill patients [37]. Additionally, the SOFA score has shown higher specificity in predicting ICU hospitalization rates and mortality in SAP patients compared to the APACHE II score [38]. The nomogram developed in this study demonstrated comparable predictive performance for hospital mortality in elderly patients compared to the SOFA score. Moreover, DCA was used to assess the clinical effectiveness of nomogram-guided medical interventions for elderly patients with SAP. The findings indicated that nomogram-guided interventions yielded greater net benefits than the SOFA score when the threshold probability ranged from 0.2 to 0.8.

Our study still has some limitations. Firstly, this study was retrospective, which means that selection and detection bias could exist. To improve the level of evidence, prospective studies are needed. Secondly, this was a single-center retrospective study that was only internally validated and lacked external validation. Therefore, our findings will need to be confirmed through studies on larger, multi-center cohorts. Thirdly, the nomogram only considers examination indicators at admission, not the dynamic changes in these indicators. Clinicians usually assess the patient’s condition sequentially and modify treatment strategies accordingly. Finally, despite our efforts to adjust for confounding factors through multivariate logistic regression analysis, there may still be residual confounding factors from unknown or unmeasured covariates that have not been completely ruled out.

## Conclusion

A nomogram model was developed for elderly patients, incorporating clinical and laboratory parameters measured upon admission to predict SAP mortality accurately. This tool shows promise in aiding clinicians to tailor individualized treatment plans for these patients, ultimately enhancing patient outcomes, conserving medical resources and costs, and facilitating early recovery.

## Data Availability

The data sets used during the study are available from the corresponding author on reasonable request.

## Abbreviations

AP: Acute pancreatitis
SAP: Severe acute pancreatitis
SOFA: Sequential organ failure assessment
APACHE-II: Acute Physiology and Chronic Health Evaluation II
ICU: Intensive care unit
RR: Respiratory rate
HR: Heart rate
SBP: Systolic blood pressure
DBP: Diastolic blood pressure
POS_2_: Arterial oxygen partial pressure
PACo_2_: Arterial carbon dioxide partial pressure
LASSO: Least absolute shrinkage and selection operator
AUC: Area under the receiver operating characteristic curve
NRI: Net reclassification improvement
IDI: Integrated discrimination improvement
DCA: Decision-curve analysis
ARF: Acute respiratory failure
